# Supplementary material for: A fault-intrusion-tolerant system and deadline-aware algorithm for scheduling scientific workflow in the cloud
Source: PeerJ Comput Sci. 2021 Nov 2;7:e747. doi: 10.7717/peerj-cs.747 (PMC8576554; doi:10.7717/peerj-cs.747)
Supplement: Supplemental Information 6 [file peerj-cs-07-747-s006.zip › Myproject1.1/docs/overview-frame.html]

Overview List


All Classes

## Packages

- org.cloudbus.cloudsim
- org.cloudbus.cloudsim.core
- org.cloudbus.cloudsim.core.predicates
- org.cloudbus.cloudsim.distributions
- org.cloudbus.cloudsim.examples
- org.cloudbus.cloudsim.examples.network
- org.cloudbus.cloudsim.examples.network.datacenter
- org.cloudbus.cloudsim.examples.power
- org.cloudbus.cloudsim.examples.power.planetlab
- org.cloudbus.cloudsim.examples.power.random
- org.cloudbus.cloudsim.lists
- org.cloudbus.cloudsim.network
- org.cloudbus.cloudsim.network.datacenter
- org.cloudbus.cloudsim.power
- org.cloudbus.cloudsim.power.lists
- org.cloudbus.cloudsim.power.models
- org.cloudbus.cloudsim.provisioners
- org.cloudbus.cloudsim.util
- org.workflowsim
- org.workflowsim.clustering
- org.workflowsim.clustering.balancing
- org.workflowsim.clustering.balancing.methods
- org.workflowsim.clustering.balancing.metrics
- org.workflowsim.examples
- org.workflowsim.failure
- org.workflowsim.planning
- org.workflowsim.reclustering
- org.workflowsim.scheduler
- org.workflowsim.utils
